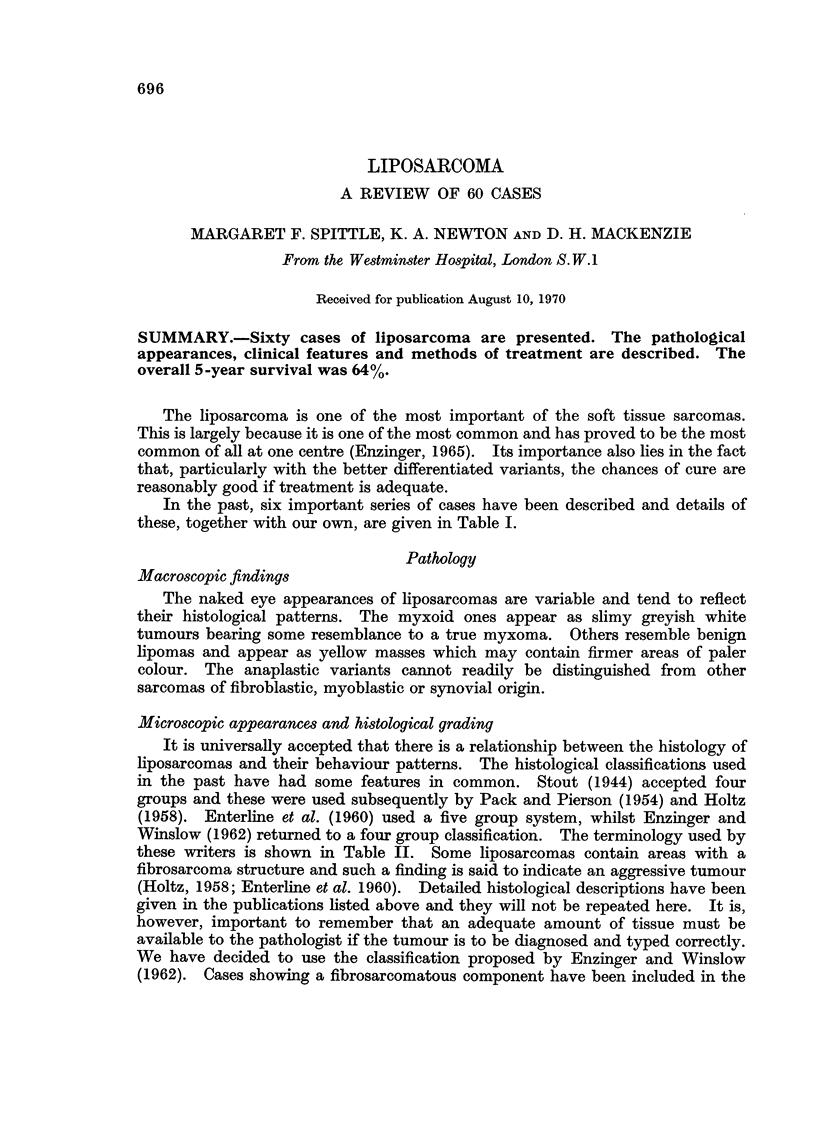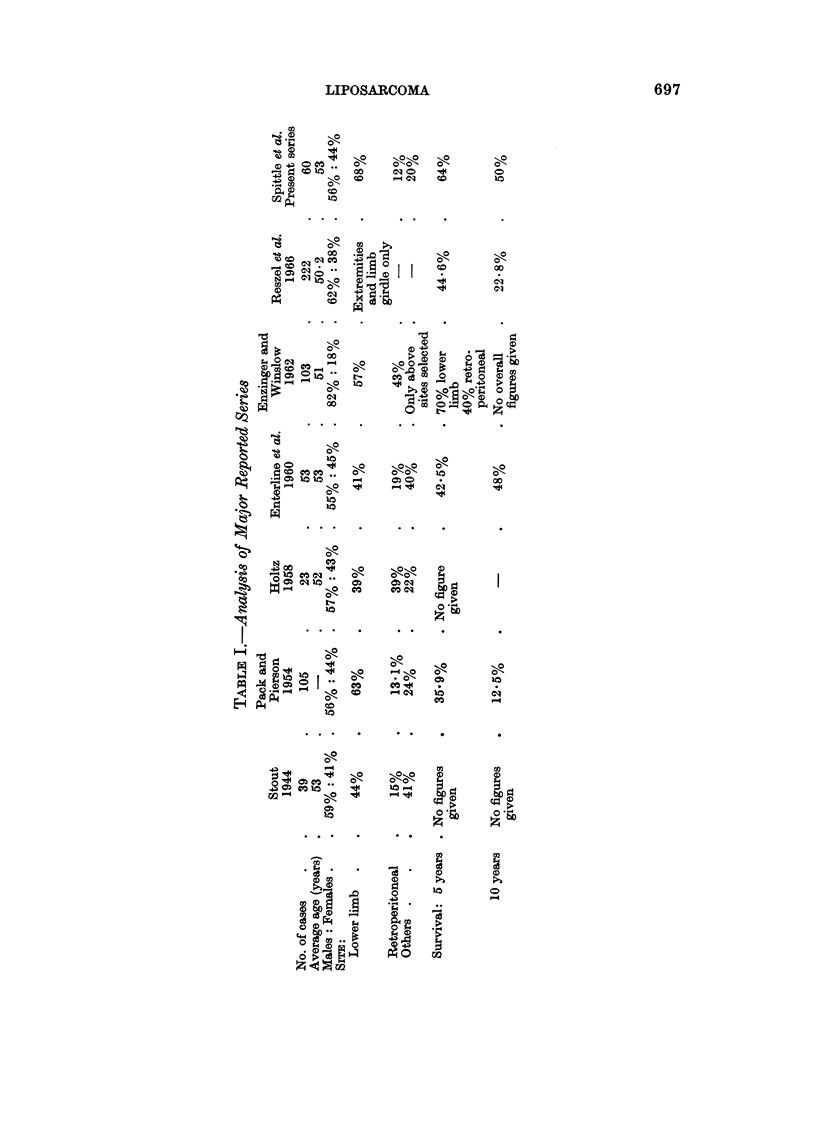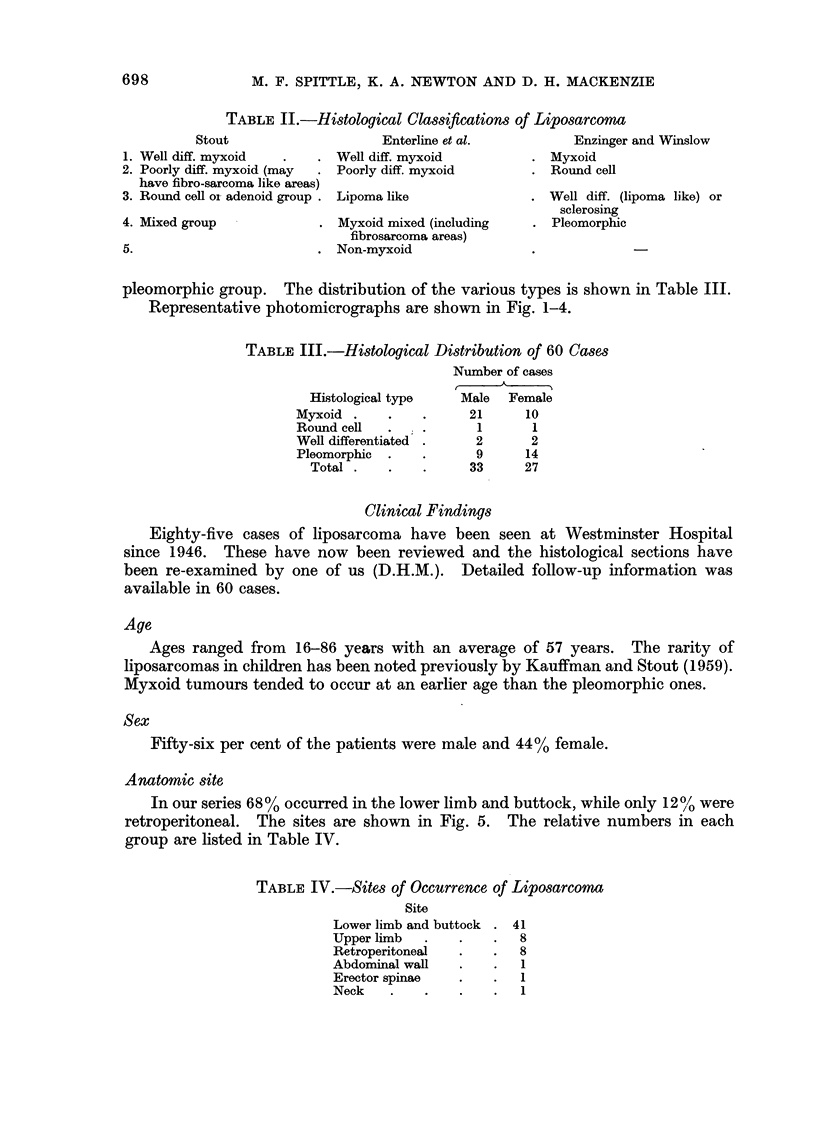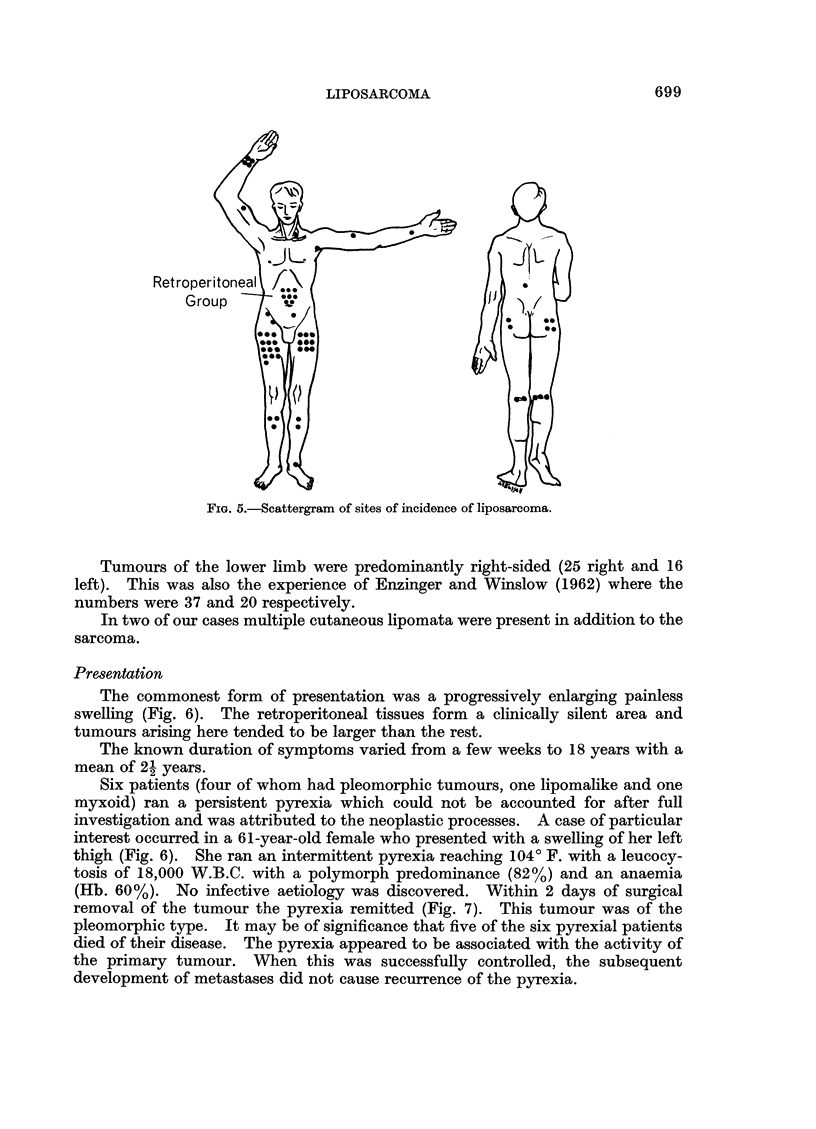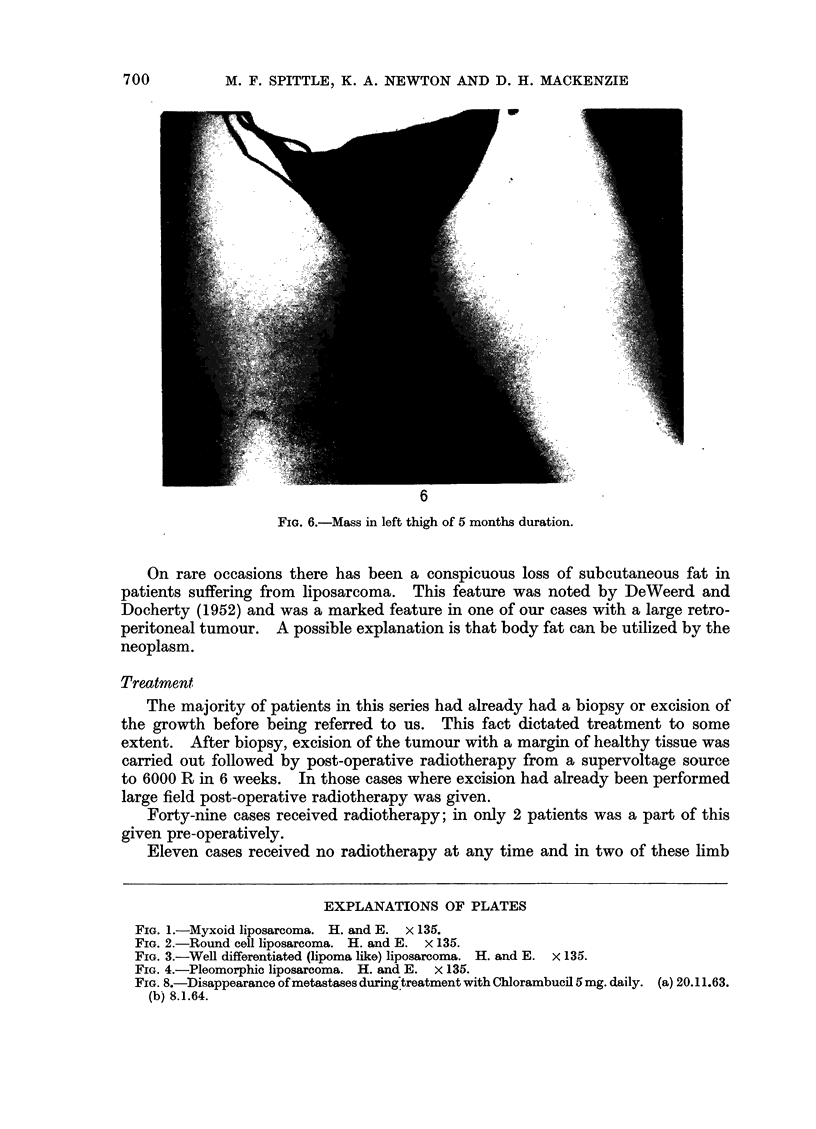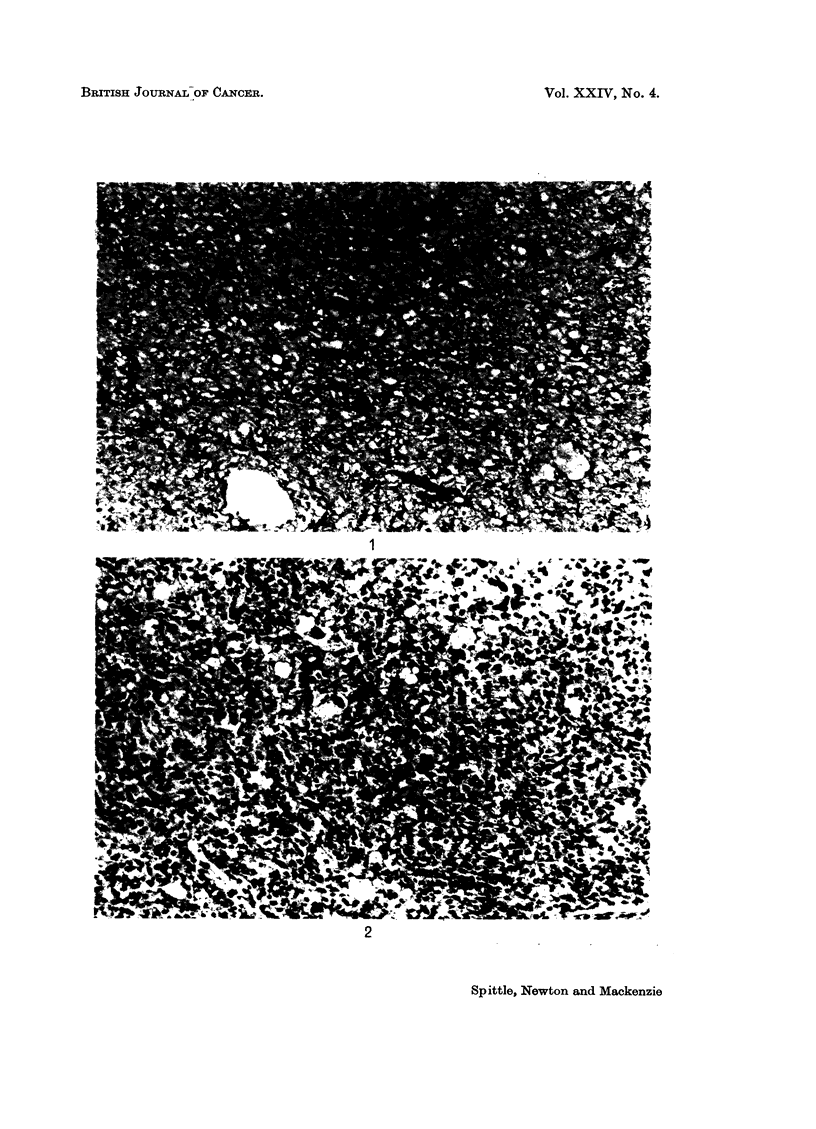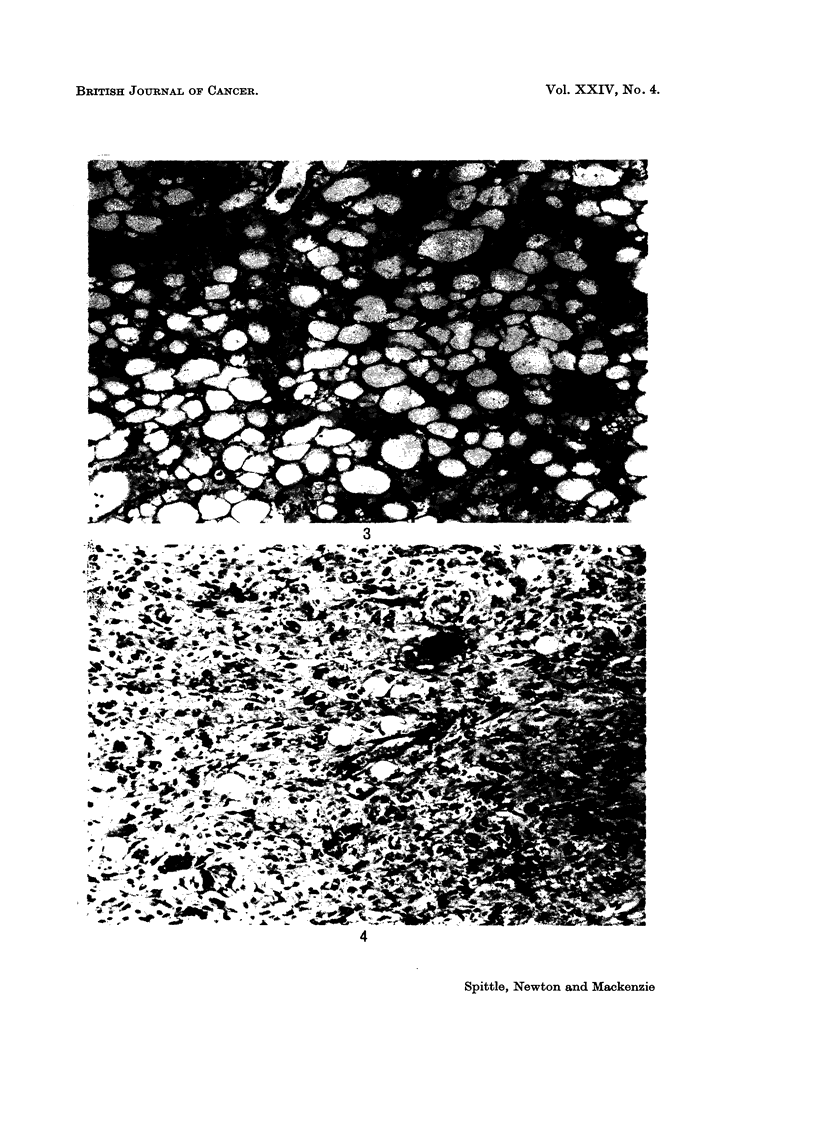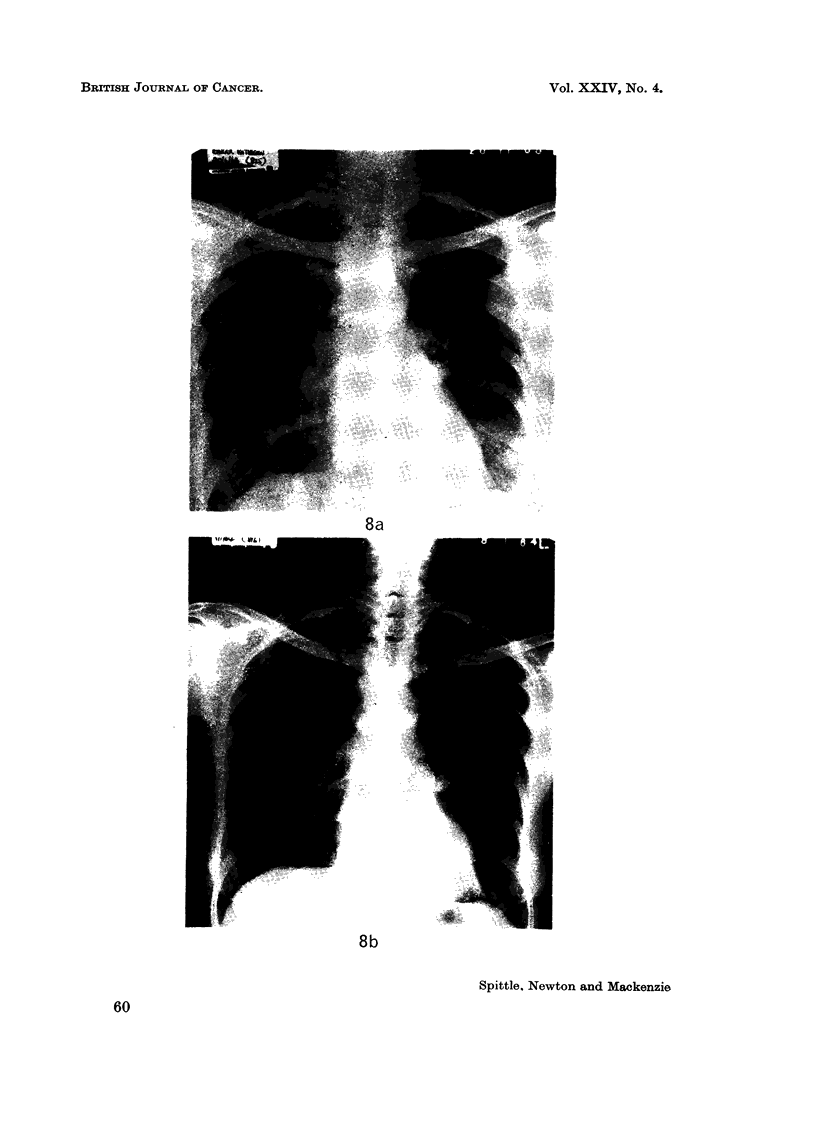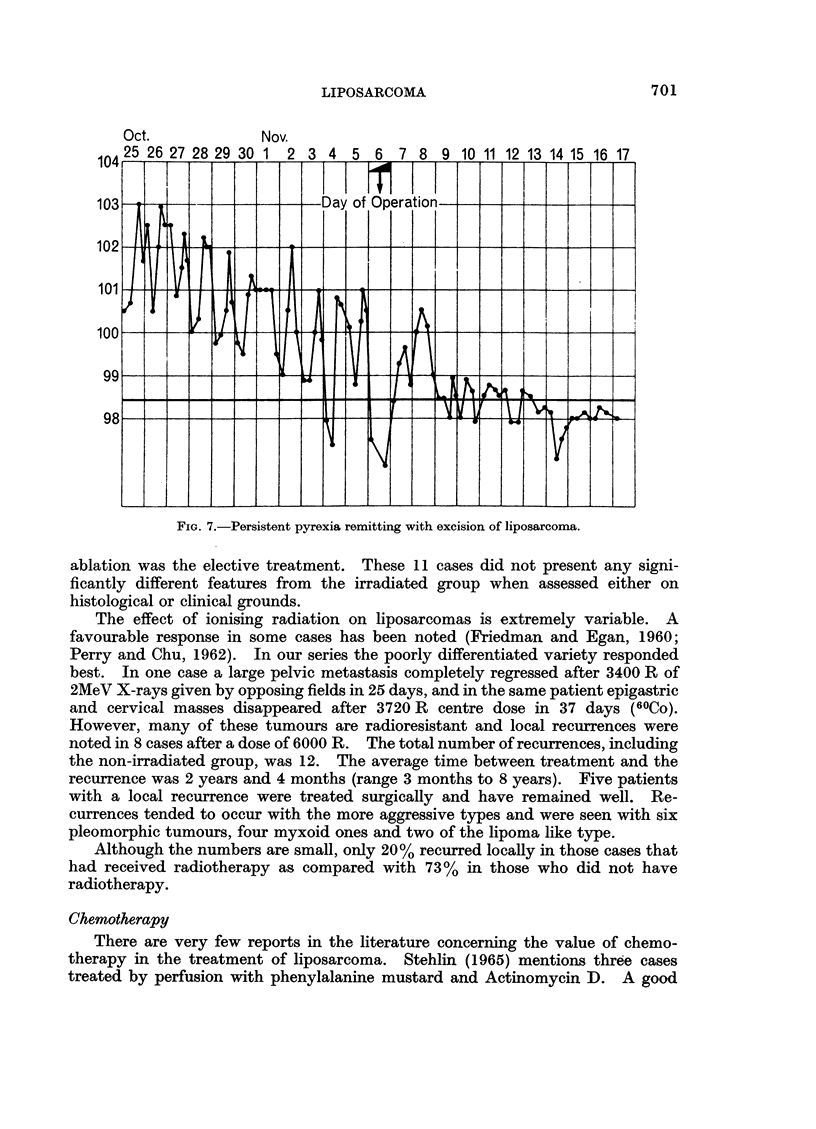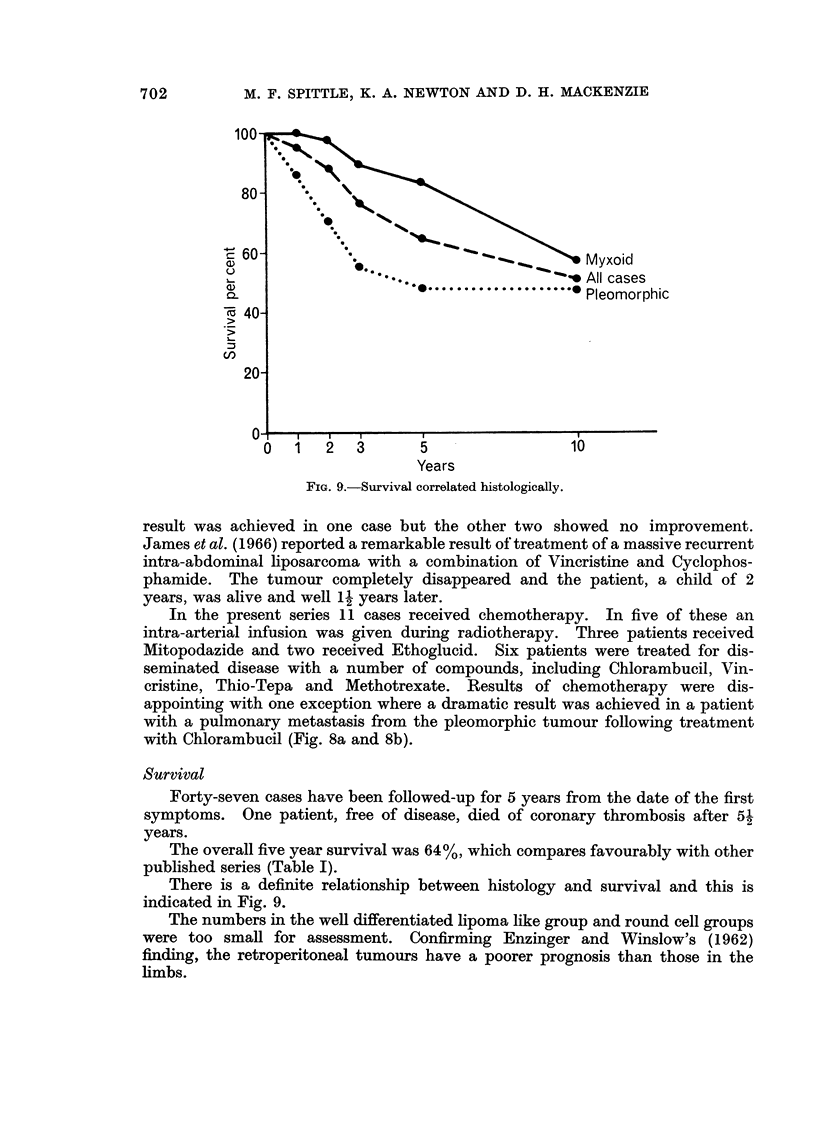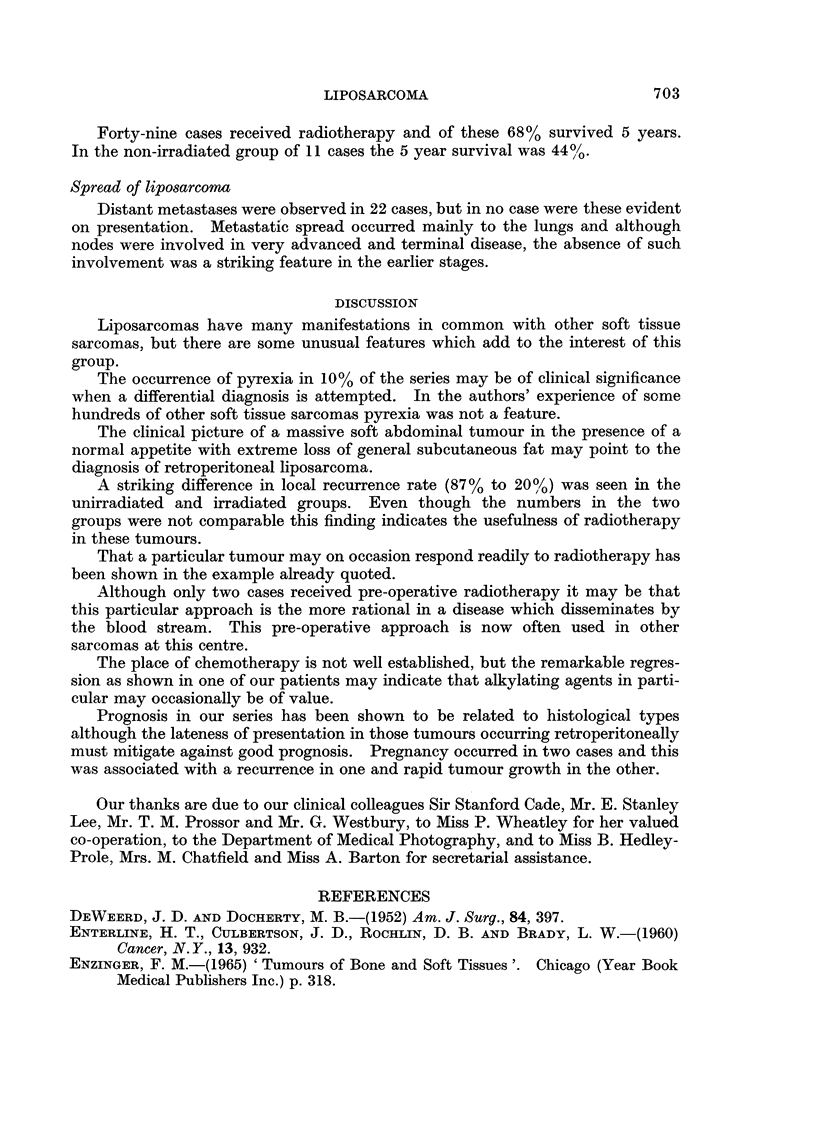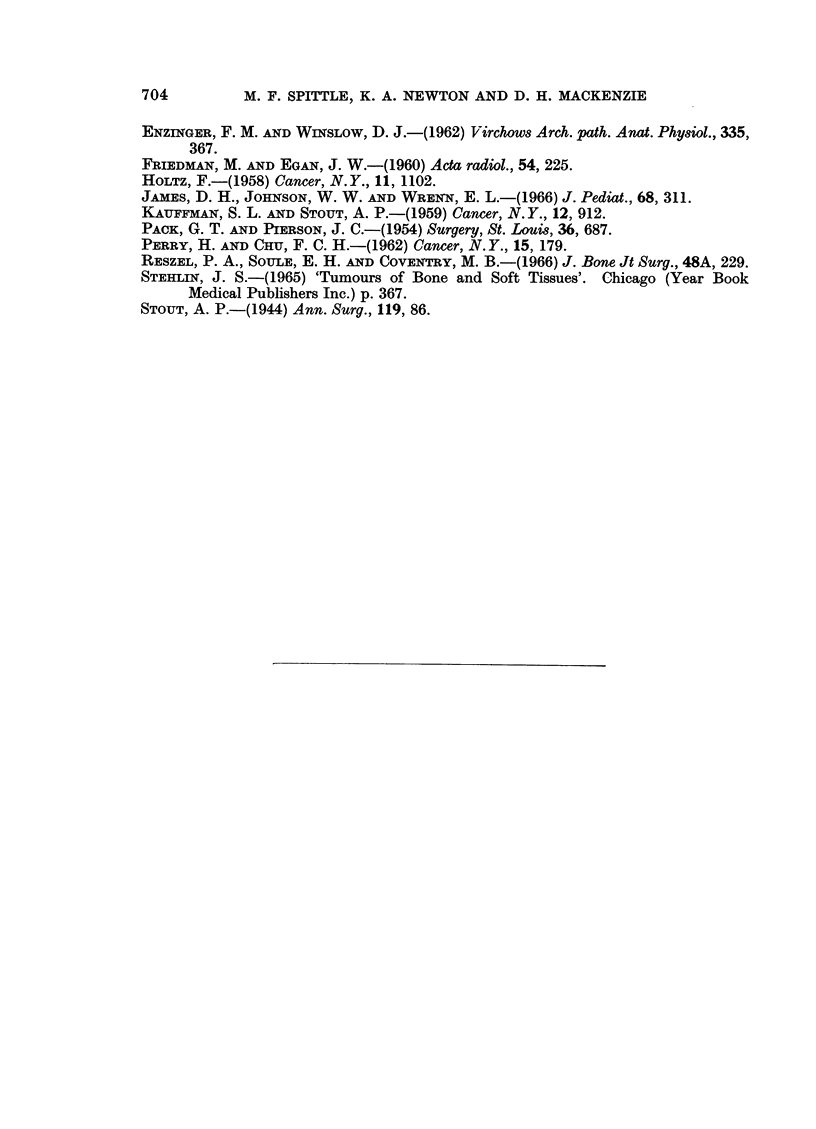# Liposarcoma

**DOI:** 10.1038/bjc.1970.83

**Published:** 1970-12

**Authors:** Margaret F. Spittle, K. A. Newton, D. H. Mackenzie

## Abstract

**Images:**


### LIPOSARCOMA

### A REVIEW OF 60 CASES

MARGARET F. SPITTLE, K. A. NEWTON AND D. H. MACKENZIE

*From the Westminster Hospital, London S.W.1*

Received for publication August 10, 1970

**SUMMARY.—Sixty cases of liposarcoma are presented. The pathological appearances, clinical features and methods of treatment are described. The overall 5-year survival was 64%.**

The liposarcoma is one of the most important of the soft tissue sarcomas. This is largely because it is one of the most common and has proved to be the most common of all at one centre (Enzinger, 1965). Its importance also lies in the fact that, particularly with the better differentiated variants, the chances of cure are reasonably good if treatment is adequate.

In the past, six important series of cases have been described and details of these, together with our own, are given in Table I.

### *Pathology*

#### *Macroscopic findings*

The naked eye appearances of liposarcomas are variable and tend to reflect their histological patterns. The myxoid ones appear as slimy greyish white tumours bearing some resemblance to a true myxoma. Others resemble benign lipomas and appear as yellow masses which may contain firmer areas of paler colour. The anaplastic variants cannot readily be distinguished from other sarcomas of fibroblastic, myoblastic or synovial origin.

#### *Microscopic appearances and histological grading*

It is universally accepted that there is a relationship between the histology of liposarcomas and their behaviour patterns. The histological classifications used in the past have had some features in common. Stout (1944) accepted four groups and these were used subsequently by Pack and Pierson (1954) and Holtz (1958). Enterline *et al.* (1960) used a five group system, whilst Enzinger and Winslow (1962) returned to a four group classification. The terminology used by these writers is shown in Table II. Some liposarcomas contain areas with a fibrosarcoma structure and such a finding is said to indicate an aggressive tumour (Holtz, 1958; Enterline *et al.* 1960). Detailed histological descriptions have been given in the publications listed above and they will not be repeated here. It is, however, important to remember that an adequate amount of tissue must be available to the pathologist if the tumour is to be diagnosed and typed correctly. We have decided to use the classification proposed by Enzinger and Winslow (1962). Cases showing a fibrosarcomatous component have been included in the

TABLE I.—*Analysis of Major Reported Series*

| | Stout 1944 | Pack and Pierson 1954 | Holtz 1958 | Enterline *et al.* 1960 | Enzinger and Winslow 1962 | Reszel *et al.* 1966 | Spittle *et al.* Present series |
|---|---|---|---|---|---|---|---|
| No. of cases | 39 | 105 | 23 | 53 | 103 | 222 | 60 |
| Average age (years) | 53 | — | 52 | 53 | 51 | 50·2 | 53 |
| Males : Females | 59% : 41% | 56% : 44% | 57% : 43% | 55% : 45% | 82% : 18% | 62% : 38% | 56% : 44% |
| SITE: | | | | | | | |
| Lower limb | 44% | 63% | 39% | 41% | 57% | Extremities and limb girdle only | 68% |
| Retroperitoneal | 15% | 13·1% | 39% | 19% | 43% | — | 12% |
| Others | 41% | 24% | 22% | 40% | Only above sites selected | — | 20% |
| Survival: 5 years | No figures given | 35·9% | No figure given | 42·5% | 70% lower limb<br>40% retro-peritoneal | 44·6% | 64% |
| 10 years | No figures given | 12·5% | — | 48% | No overall figures given | 22·8% | 50% |

TABLE II.—*Histological Classifications of Liposarcoma*

| Stout | Enterline *et al.* | Enzinger and Winslow |
|---|---|---|
| 1. Well diff. myxoid | Well diff. myxoid | Myxoid |
| 2. Poorly diff. myxoid (may have fibro-sarcoma like areas) | Poorly diff. myxoid | Round cell |
| 3. Round cell or adenoid group | Lipoma like | Well diff. (lipoma like) or sclerosing |
| 4. Mixed group | Myxoid mixed (including fibrosarcoma areas) | Pleomorphic |
| 5. | Non-myxoid | — |

pleomorphic group. The distribution of the various types is shown in Table III.

Representative photomicrographs are shown in Fig. 1–4.

TABLE III.—*Histological Distribution of* 60 *Cases*

| | Number of cases | |
|---|---|---|
| Histological type | Male | Female |
| Myxoid | 21 | 10 |
| Round cell | 1 | 1 |
| Well differentiated | 2 | 2 |
| Pleomorphic | 9 | 14 |
| Total | 33 | 27 |

### *Clinical Findings*

Eighty-five cases of liposarcoma have been seen at Westminster Hospital since 1946. These have now been reviewed and the histological sections have been re-examined by one of us (D.H.M.). Detailed follow-up information was available in 60 cases.

### *Age*

Ages ranged from 16–86 years with an average of 57 years. The rarity of liposarcomas in children has been noted previously by Kauffman and Stout (1959). Myxoid tumours tended to occur at an earlier age than the pleomorphic ones.

### *Sex*

Fifty-six per cent of the patients were male and 44% female.

### *Anatomic site*

In our series 68% occurred in the lower limb and buttock, while only 12% were retroperitoneal. The sites are shown in Fig. 5. The relative numbers in each group are listed in Table IV.

TABLE IV.—*Sites of Occurrence of Liposarcoma*

| Site | |
|---|---|
| Lower limb and buttock | 41 |
| Upper limb | 8 |
| Retroperitoneal | 8 |
| Abdominal wall | 1 |
| Erector spinae | 1 |
| Neck | 1 |

FIG. 5.—Scattergram of sites of incidence of liposarcoma.

Tumours of the lower limb were predominantly right-sided (25 right and 16 left). This was also the experience of Enzinger and Winslow (1962) where the numbers were 37 and 20 respectively.

In two of our cases multiple cutaneous lipomata were present in addition to the sarcoma.

*Presentation*

The commonest form of presentation was a progressively enlarging painless swelling (Fig. 6). The retroperitoneal tissues form a clinically silent area and tumours arising here tended to be larger than the rest.

The known duration of symptoms varied from a few weeks to 18 years with a mean of $2\frac{1}{2}$ years.

Six patients (four of whom had pleomorphic tumours, one lipomalike and one myxoid) ran a persistent pyrexia which could not be accounted for after full investigation and was attributed to the neoplastic processes. A case of particular interest occurred in a 61-year-old female who presented with a swelling of her left thigh (Fig. 6). She ran an intermittent pyrexia reaching 104° F. with a leucocytosis of 18,000 W.B.C. with a polymorph predominance (82%) and an anaemia (Hb. 60%). No infective aetiology was discovered. Within 2 days of surgical removal of the tumour the pyrexia remitted (Fig. 7). This tumour was of the pleomorphic type. It may be of significance that five of the six pyrexial patients died of their disease. The pyrexia appeared to be associated with the activity of the primary tumour. When this was successfully controlled, the subsequent development of metastases did not cause recurrence of the pyrexia.

FIG. 6.—Mass in left thigh of 5 months duration.

On rare occasions there has been a conspicuous loss of subcutaneous fat in patients suffering from liposarcoma. This feature was noted by DeWeerd and Docherty (1952) and was a marked feature in one of our cases with a large retroperitoneal tumour. A possible explanation is that body fat can be utilized by the neoplasm.

*Treatment*

The majority of patients in this series had already had a biopsy or excision of the growth before being referred to us. This fact dictated treatment to some extent. After biopsy, excision of the tumour with a margin of healthy tissue was carried out followed by post-operative radiotherapy from a supervoltage source to 6000 R in 6 weeks. In those cases where excision had already been performed large field post-operative radiotherapy was given.

Forty-nine cases received radiotherapy; in only 2 patients was a part of this given pre-operatively.

Eleven cases received no radiotherapy at any time and in two of these limb

---

EXPLANATIONS OF PLATES

FIG. 1.—Myxoid liposarcoma. H. and E. × 135.
FIG. 2.—Round cell liposarcoma. H. and E. × 135.
FIG. 3.—Well differentiated (lipoma like) liposarcoma. H. and E. × 135.
FIG. 4.—Pleomorphic liposarcoma. H. and E. × 135.
FIG. 8.—Disappearance of metastases during treatment with Chlorambucil 5 mg. daily. (a) 20.11.63. (b) 8.1.64.

1

2

Spittle, Newton and Mackenzie

3

4

Spittle, Newton and Mackenzie

8a

8b

Spittle, Newton and Mackenzie

FIG. 7.—Persistent pyrexia remitting with excision of liposarcoma.

ablation was the elective treatment. These 11 cases did not present any significantly different features from the irradiated group when assessed either on histological or clinical grounds.

The effect of ionising radiation on liposarcomas is extremely variable. A favourable response in some cases has been noted (Friedman and Egan, 1960; Perry and Chu, 1962). In our series the poorly differentiated variety responded best. In one case a large pelvic metastasis completely regressed after 3400 R of 2MeV X-rays given by opposing fields in 25 days, and in the same patient epigastric and cervical masses disappeared after 3720 R centre dose in 37 days ($^{60}Co$). However, many of these tumours are radioresistant and local recurrences were noted in 8 cases after a dose of 6000 R. The total number of recurrences, including the non-irradiated group, was 12. The average time between treatment and the recurrence was 2 years and 4 months (range 3 months to 8 years). Five patients with a local recurrence were treated surgically and have remained well. Recurrences tended to occur with the more aggressive types and were seen with six pleomorphic tumours, four myxoid ones and two of the lipoma like type.

Although the numbers are small, only 20% recurred locally in those cases that had received radiotherapy as compared with 73% in those who did not have radiotherapy.

*Chemotherapy*

There are very few reports in the literature concerning the value of chemotherapy in the treatment of liposarcoma. Stehlin (1965) mentions three cases treated by perfusion with phenylalanine mustard and Actinomycin D. A good

FIG. 9.—Survival correlated histologically.

result was achieved in one case but the other two showed no improvement. James *et al.* (1966) reported a remarkable result of treatment of a massive recurrent intra-abdominal liposarcoma with a combination of Vincristine and Cyclophosphamide. The tumour completely disappeared and the patient, a child of 2 years, was alive and well 1½ years later.

In the present series 11 cases received chemotherapy. In five of these an intra-arterial infusion was given during radiotherapy. Three patients received Mitopodazide and two received Ethoglucid. Six patients were treated for disseminated disease with a number of compounds, including Chlorambucil, Vincristine, Thio-Tepa and Methotrexate. Results of chemotherapy were disappointing with one exception where a dramatic result was achieved in a patient with a pulmonary metastasis from the pleomorphic tumour following treatment with Chlorambucil (Fig. 8a and 8b).

*Survival*

Forty-seven cases have been followed-up for 5 years from the date of the first symptoms. One patient, free of disease, died of coronary thrombosis after 5½ years.

The overall five year survival was 64%, which compares favourably with other published series (Table I).

There is a definite relationship between histology and survival and this is indicated in Fig. 9.

The numbers in the well differentiated lipoma like group and round cell groups were too small for assessment. Confirming Enzinger and Winslow's (1962) finding, the retroperitoneal tumours have a poorer prognosis than those in the limbs.

Forty-nine cases received radiotherapy and of these 68% survived 5 years. In the non-irradiated group of 11 cases the 5 year survival was 44%.

*Spread of liposarcoma*

Distant metastases were observed in 22 cases, but in no case were these evident on presentation. Metastatic spread occurred mainly to the lungs and although nodes were involved in very advanced and terminal disease, the absence of such involvement was a striking feature in the earlier stages.

### DISCUSSION

Liposarcomas have many manifestations in common with other soft tissue sarcomas, but there are some unusual features which add to the interest of this group.

The occurrence of pyrexia in 10% of the series may be of clinical significance when a differential diagnosis is attempted. In the authors' experience of some hundreds of other soft tissue sarcomas pyrexia was not a feature.

The clinical picture of a massive soft abdominal tumour in the presence of a normal appetite with extreme loss of general subcutaneous fat may point to the diagnosis of retroperitoneal liposarcoma.

A striking difference in local recurrence rate (87% to 20%) was seen in the unirradiated and irradiated groups. Even though the numbers in the two groups were not comparable this finding indicates the usefulness of radiotherapy in these tumours.

That a particular tumour may on occasion respond readily to radiotherapy has been shown in the example already quoted.

Although only two cases received pre-operative radiotherapy it may be that this particular approach is the more rational in a disease which disseminates by the blood stream. This pre-operative approach is now often used in other sarcomas at this centre.

The place of chemotherapy is not well established, but the remarkable regression as shown in one of our patients may indicate that alkylating agents in particular may occasionally be of value.

Prognosis in our series has been shown to be related to histological types although the lateness of presentation in those tumours occurring retroperitoneally must mitigate against good prognosis. Pregnancy occurred in two cases and this was associated with a recurrence in one and rapid tumour growth in the other.

Our thanks are due to our clinical colleagues Sir Stanford Cade, Mr. E. Stanley Lee, Mr. T. M. Prossor and Mr. G. Westbury, to Miss P. Wheatley for her valued co-operation, to the Department of Medical Photography, and to Miss B. Hedley-Prole, Mrs. M. Chatfield and Miss A. Barton for secretarial assistance.

### REFERENCES

DeWeerd, J. D. and Docherty, M. B.—(1952) *Am. J. Surg.*, **84**, 397.

Enterline, H. T., Culbertson, J. D., Rochlin, D. B. and Brady, L. W.—(1960) *Cancer, N.Y.*, **13**, 932.

Enzinger, F. M.—(1965) 'Tumours of Bone and Soft Tissues'. Chicago (Year Book Medical Publishers Inc.) p. 318.

ENZINGER, F. M. AND WINSLOW, D. J.—(1962) *Virchows Arch. path. Anat. Physiol.*, **335**, 367.
FRIEDMAN, M. AND EGAN, J. W.—(1960) *Acta radiol.*, **54**, 225.
HOLTZ, F.—(1958) *Cancer, N.Y.*, **11**, 1102.
JAMES, D. H., JOHNSON, W. W. AND WRENN, E. L.—(1966) *J. Pediat.*, **68**, 311.
KAUFFMAN, S. L. AND STOUT, A. P.—(1959) *Cancer, N.Y.*, **12**, 912.
PACK, G. T. AND PIERSON, J. C.—(1954) *Surgery, St. Louis*, **36**, 687.
PERRY, H. AND CHU, F. C. H.—(1962) *Cancer, N.Y.*, **15**, 179.
RESZEL, P. A., SOULE, E. H. AND COVENTRY, M. B.—(1966) *J. Bone Jt Surg.*, **48**A, 229.
STEHLIN, J. S.—(1965) 'Tumours of Bone and Soft Tissues'. Chicago (Year Book Medical Publishers Inc.) p. 367.
STOUT, A. P.—(1944) *Ann. Surg.*, **119**, 86.